# The Role of PAR2 in TGF-β1-Induced ERK Activation and Cell Motility

**DOI:** 10.3390/ijms18122776

**Published:** 2017-12-20

**Authors:** Hendrik Ungefroren, David Witte, Christian Fiedler, Thomas Gädeken, Roland Kaufmann, Hendrik Lehnert, Frank Gieseler, Bernhard H. Rauch

**Affiliations:** 1 First Department of Medicine, UKSH, Campus Lübeck, 23538 Lübeck, Germany; davidwittemail@gmail.com (D.W.); c.fiedler93@gmx.de (C.F.); T.Gaedeken@web.de (T.G.); Hendrik.Lehnert@uni-luebeck.de (H.L.); frank.gieseler@uksh.de (F.G.); 2 Department of General and Thoracic Surgery, UKSH, Campus Kiel, 24105 Kiel, Germany; 3 Department of General, Visceral and Vascular Surgery, Jena University Hospital, 07747 Jena, Germany; Roland.Kaufmann@med.uni-jena.de; 4 Institute of Pharmacology, Department of General Pharmacology, University Medicine Greifswald, 17487 Greifswald, Germany; Bernhard.Rauch@uni-greifswald.de

**Keywords:** ALK5, ERK, cell migration, PAR2, SMAD, transforming growth factor-β

## Abstract

Background: Recently, the expression of proteinase-activated receptor 2 (PAR2) has been shown to be essential for activin receptor-like kinase 5 (ALK5)/SMAD-mediated signaling and cell migration by transforming growth factor (TGF)-β1. However, it is not known whether activation of non-SMAD TGF-β signaling (e.g., RAS–RAF–MEK–extracellular signal-regulated kinase (ERK) signaling) is required for cell migration and whether it is also dependent on PAR2. Methods: RNA interference was used to deplete cells of PAR2, followed by xCELLigence technology to measure cell migration, phospho-immunoblotting to assess ERK1/2 activation, and co-immunoprecipitation to detect a PAR2–ALK5 physical interaction. Results: Inhibition of ERK signaling with the MEK inhibitor U0126 blunted the ability of TGF-β1 to induce migration in pancreatic cancer Panc1 cells. ERK activation in response to PAR2 agonistic peptide (PAR2–AP) was strong and rapid, while it was moderate and delayed in response to TGF-β1. Basal and TGF-β1-dependent ERK, but not SMAD activation, was blocked by U0126 in Panc1 and other cell types indicating that ERK activation is downstream or independent of SMAD signaling. Moreover, cellular depletion of PAR2 in HaCaT cells strongly inhibited TGF-β1-induced ERK activation, while the biased PAR2 agonist GB88 at 10 and 100 µM potentiated TGF-β1-dependent ERK activation and cell migration. Finally, we provide evidence for a physical interaction between PAR2 and ALK5. Our data show that both PAR2–AP- and TGF-β1-induced cell migration depend on ERK activation, that PAR2 expression is crucial for TGF-β1-induced ERK activation, and that the functional cooperation of PAR2 and TGF-β1 involves a physical interaction between PAR2 and ALK5.

## 1. Introduction

Transforming growth factor (TGF)-β1 controls many cellular functions under physiological conditions and in disease states such as fibrosis and cancer. TGF-β resides in the tumor microenvironment in a latent form and upon activation binds to its cognate receptor(s) on target cells to control proliferation, cell motility, and morphological plasticity. TGF-β signals through two transmembrane serine/threonine kinase receptors termed type II and type I/activin receptor-like kinase 5 (ALK5). ALK5 activates the canonical SMAD pathway by C-terminal phosphorylation of SMAD2 and SMAD3. Activated SMAD2/3 binds to SMAD4 and the complex is translocated to the nucleus to control transcriptional activity of TGF-β target genes [1]. The ligand–receptor complex may also trigger non-SMAD pathways; for example, MKK6–p38 and MEK–extracellular signal-regulated kinase (ERK) mitogen-activated protein kinases (MAPK) [1]. These non-SMAD pathways cooperate with SMAD signaling to regulate epithelial-to-mesenchymal transition (EMT) [2,3], migration, invasion, and metastasis [4,5,6,7]. In both normal and malignant Kirsten RAS (KRAS)-expressing pancreas, *TGFBR2* and *TGFBR1* (encoding ALK5) are necessary for ERK phosphorylation in vivo. Notably, ALK5 was found to possess tyrosine kinase activity and to directly phosphorylate ShcA resulting in activation of MEK/ERK signaling in Mv1Lu mink epithelial cells and in 3T3-Swiss cells [8,9]. Additionally, ERK has been shown to antagonize TGF-β canonical SMAD signaling through phosphorylation of SMAD2 and SMAD3 within the SMAD-linker region and inhibiting SMAD2/3 signaling [10], while enhancement of SMAD signaling can occur by ERK-mediated phosphorylation at other SMAD3 residues (e.g., Thr8).

A recent report suggests that during carcinogenesis, cross-talk with the RAS–MEK–ERK signaling cascade initially facilitates and later antagonizes TGF-β-mediated cell cycle arrest as evidenced by upregulation of growth-suppressive phospho-SMAD2 and p21 [9]. This study also showed that RAS–MEK–ERK signaling remains crucial for the EMT-inducing arm of TGF-β signaling in both benign and malignant cells as determined by morphology and EMT-marker expression [9]. However, whether ERK signaling is also required for other EMT-associated functions such as cell migration and invasion in cancer cells remains to be elucidated. In TGF-β-responsive pancreatic cancer cell lines such as Panc1, TGF-β1 has been shown to induce a moderate but sustained activation of ERK2, which was required for TGF-β-dependent invasion [11,12].

Proteinase-activated receptor 2 (PAR2) belongs to a subgroup of G protein-coupled receptors that comprise PAR1–PAR4 [13,14,15]. These receptors are characterized by a unique mechanism of proteolytic activation through serine proteinases that cleave the receptor at specific recognition sites within the extracellular N-terminus. Cleavage leads to the exposure of a short amino-terminal sequence (“tethered ligand”) that remains covalently linked to the receptor and binds to the extracellular part of the receptor to induce a change in conformation and activation of signaling from G proteins, β-arrestin, and eventually transactivation of various other receptors [13,14,15]. PAR2 is cleaved by trypsin, which generates signaling via multiple G proteins (G_q_/G_12/13_) and β-arrestin to signal via elevated intracellular calcium and ERK MAPK [13,14]. ERK may also be activated via “non-canonical” cleavage of the PAR2 N-terminal domain (e.g., by neutrophil elastase in “biased signaling”) to activate by a process that is G_12/13_-Rho kinase-dependent, but both calcium- and β-arrestin-independent [16,17]. ERK activation by PAR2 has important physiological roles in the nervous system [18,19,20] and in PAR2-stimulated cell migration [21,22,23].

Recently, we revealed in cells derived from pancreatic ductal adenocarcinoma (PDAC) and in the immortalized keratinocyte cell line HaCaT a novel mode of interaction between TGF-β1/ALK5 and PAR2 signaling. Specifically, PAR2 protein was required for maintaining expression of ALK5 and ALK5-mediated pro-oncogenic effects of TGF-β1 such as migration and invasion [24,25,26]. Conversely, however, ALK5 was not required for PAR2–AP-stimulated cell migration [26]. These data show that while TGF-β1-mediated cell migration depends on both ALK5 and PAR2, migration triggered by the activation of PAR2 with either PAR2–AP or trypsin is ALK5-independent. As outlined above, MEK–ERK signaling may be stimulated directly via the tyrosine kinase function of ALK5. However, ALK5 may also be able to activate ERK by transactivation of other receptors that stimulate ERK1/2, such as epidermal growth factor receptor (EGFR) [27] or PAR2. In turn, PAR2-induced activation of ERK in HaCaT keratinocytes required transactivation of EGFR [28]. Moreover, stimulation of HaCaT cells with trypsin strongly increased the secretion of TGF-β1 and this effect was fully dependent on PAR2-mediated EGFR transactivation as revealed by its sensitivity to MEK1 inhibition [28]. Finally, PAR2 has been shown to transactivate EGFR and TGF-β receptor signaling in connective tissue growth factor (CTGF) expression in renal cells [29]. These data point to a complex and intimate receptor transactivation and autocrine signaling loop between TGF-β receptor/ALK5, PAR2, and EGFR in the regulation of RAS–MEK–ERK signaling. Prompted by the strong dependency of the migratory and many other TGF-β responses on PAR2 expression on the one hand [24], and on ERK activation on the other hand [9] in conjunction with the crucial role of ERK in PAR2-stimulated cell migration [21,22,23,30,31], we set out to test, mainly in PDAC-derived cells, whether recombinant human (rh)TGF-β1-triggered ERK activation depends on endogenous PAR2.

## 2. Results

### 2.1. ERK Activation Is Required for Both PAR2–AP- and TGF-β1-Mediated Cell Migration

**Figure 1:**
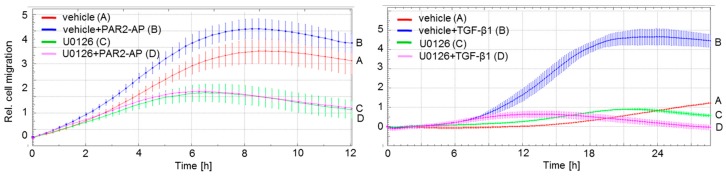
PAR2–AP- and TGF-β1-driven random cell migration are dependent on ERK activation. Panc1 cells (left-hand graph) and Colo357 cells (right-hand graph) were subjected to a cell migration assay (chemokinesis setup) in the presence of vehicle (0.1% dimethylsulfoxide, DMSO) or 20 µM U0126 and either PAR2–AP (15 µM 2-furoyl-LIGRLO-NH_2_ (2f-LI), left-hand graph) or TGF-β1 (5 ng/mL, right-hand graph). In the left graph, differences are significant (*p* < 0.05, unpaired Student’s *t*-test) between vehicle + PAR2–AP treated cells (blue curve, tracing B) and U0126 + PAR2–AP treated cells (magenta curve, tracing D) at 4:00 and all later time points, and between vehicle treated cells (red curve, tracing A) and U0126 treated cells (green curve, tracing C) at 6:00 and all later time points. In the right-hand graph, differences are significant between vehicle + TGF-β1 treated cells (blue curve, tracing B) and U0126 + TGF-β1 treated cells (magenta curve, tracing D) at 12:00 and all later time points. For a color-independent identification of the curves, see letters to the right of each graph. In each graph, data are shown from one representative experiment; three experiments were performed in total.

To assess the role of ERK activation in PAR2–AP- and TGF-β1-dependent cell motility, we subjected the PDAC cell lines Panc1 and Colo357 to real-time assays for measurement of non-directional (random) cell migration (chemokinesis setup) in the presence and absence of U0126, an inhibitor of MEK, which is the upstream kinase kinase activator of ERK1/2. Results show that U0126 potently suppressed the random migratory activity of Panc1 cells following stimulation with the PAR2–AP 2-furoyl-LIGRLO-NH_2_ (2-fLI) (Figure 1, left-hand graph) and that of Colo357 cells following stimulation with TGF-β1 (Figure 1, right-hand graph). Very similar results were obtained when a chemotaxis setup with PAR2-AP or TGF-β1 as attractants was used. The role of ERK activation in the migratory response to both agents therefore required a more thorough analysis.

### 2.2. ERK Is Activated by PAR2–AP and TGF-β1 with Different Kinetics

**Figure 2:**
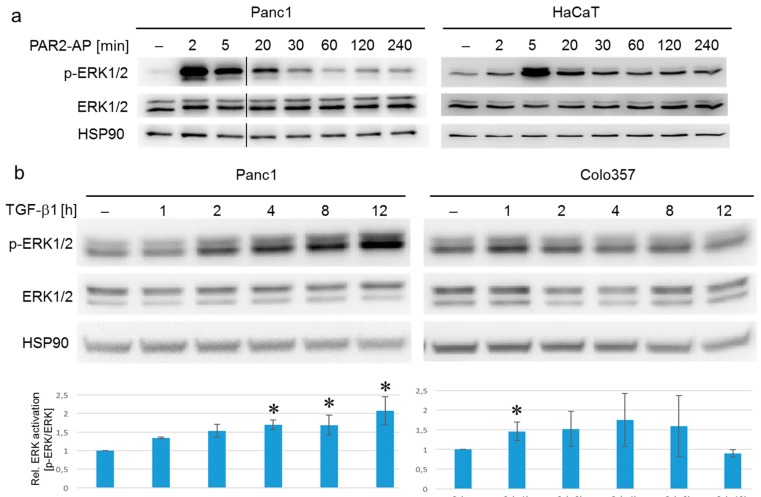
Kinetics of ERK activation in response to PAR2–AP or TGF-β1 in various cell types. (**a**) Panc1 (left) and HaCaT (right) cells were stimulated with 2f-LI (15 µM) for 2–240 min and subsequently analyzed by phospho-immunoblotting for phospho-ERK1/2 (p-ERK1/2). Following removal of the p-ERK1/2 antibody, the blot was incubated with antibodies to total ERK1/2 and to HSP90 as a loading control; (**b**) Panc1 and Colo357 cells were stimulated with TGF-β1 (5 ng/mL) for 1–12 h and subjected to immunoblot analysis for p-ERK1/2, ERK, and HSP90 as described in (**a**). The graphs below the immunoblots show the results from the densitometric analysis of four independent experiments (mean ± SD). The asterisks indicate significant differences relative to the untreated control, *p* < 0.05.

To reveal whether PAR2–AP and TGF-β1 employ the same or different downstream signal transducers to activate ERK, we measured the temporal response to both agents with respect to p-ERK1/2 accumulation in Panc1, Colo357, and HaCaT cells. ERK1/2 activation in response to PAR2-AP stimulation was rapid, peaking at 2 min after addition of the peptide and returning to background levels by 60 min (Figure 2a). ERK1 was much more strongly induced than ERK2 (Figure 2a). A similar time course was seen in HaCaT keratinocytes with a peak at 5 min (Figure 2a, and Ref. [28]). In contrast, induction of p-ERK1/2 by TGF-β1 in Panc1 cells was only moderate but sustained and was more pronounced for ERK2 with a significant increase at 4 h and another one at 12 h (Figure 2b, left). In Colo357 cells, p-ERK1/2 levels peaked at 1 h with a trend to remain elevated up to 8 h (Figure 2b, right), while HaCaT cells showed a transient peak at 1 h only [32]. Thus, ERK1/2 activation in response to TGF-β1 was delayed in all three cell lines relative to ERK1/2 activation induced by PAR2-AP. The reasons for this delay in ERK activation by TGF-β1 merit further investigation in the future.

### 2.3. ERK but Not SMAD Activation in Response to TGF-β1 Was Blocked by the MEK Inhibitor U0126

**Figure 3:**
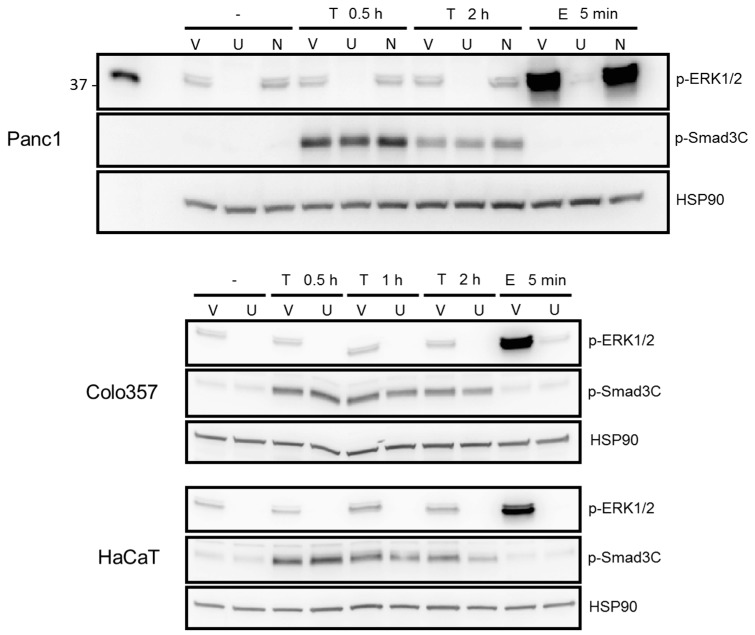
ERK1/2 but not SMAD3 activation in response to TGF-β1 was blocked by the MEK inhibitor U0126. The indicated cell lines were grown to confluence, starved for 24 h in medium containing 0.1% bovine serum albumin, and treated for the indicated times with TGF-β1 (T, 5 ng/mL) in the absence or presence of either vehicle (V, 0.2% DMSO), the MEK inhibitor U0126 (U, 20 µM), or the Rac1 inhibitor NSC23766 (N, 200 µM) as negative control. Cells were subjected to immunoblotting for p-ERK1/2, ERK1/2, p-SMAD3C, and SMAD3 and for HSP90 to control for equal loading. The total forms of ERK1/2 and SMAD3 were not different between the various time points and treatments. The functionality of U0126 was confirmed by its ability to block ERK1/2 activation after a 5 min challenge of cells with EGF (E, 10 ng/mL).

Above, we have shown that the migratory activity of Panc1 cells in response to stimulation with 2-fLI or TGF-β1 was sensitive to inhibition with U0126. To test more directly whether U0126 was able to block phosphorylation of ERK1/2, we treated control and TGF-β1- or epidermal growth factor (EGF)-treated Panc1 cells with U0126 and subjected the cells to phospho-immunoblotting. As shown in Figure 3, U0126 but neither the vehicle nor the Rac1 inhibitor NSC23766 (used as negative control) blocked basal ERK activation and that induced by treatment with TGF-β1 or EGF as positive control (Figure 3, upper panel). ERK activation can occur upstream or downstream of SMAD3 signaling. The delayed ERK activation response, which occurred later than SMAD activation (which is first detectable at approx. 15 min), might suggest that ERK activation is independent or downstream of SMAD activation. If so, inhibiting ERK should not affect TGF-β1-induced SMAD activation. Treatment of Panc1, Colo357, and HaCaT cells with a combination of U0126 and TGF-β1 failed to affect TGF-β1-induced C-terminal phosphorylation of both SMAD3C (Figure 3, middle blot in each panel) and SMAD2C. To summarize, ERK activation is crucial for PAR2–AP- and TGF-β1-induced cell motility and is induced by the respective agents with different kinetics. Moreover, TGF-β1-activated ERK was unable to interfere with SMAD2/3 C-terminal phosphorylation, which is consistent with our previous observation that PAR2–AP-mediated cell migration is ALK5 independent [26].

### 2.4. Both PAR2-AP- and TGF-β1-Induced ERK Activation are Dependent on PAR2 Protein Expression

**Figure 4:**
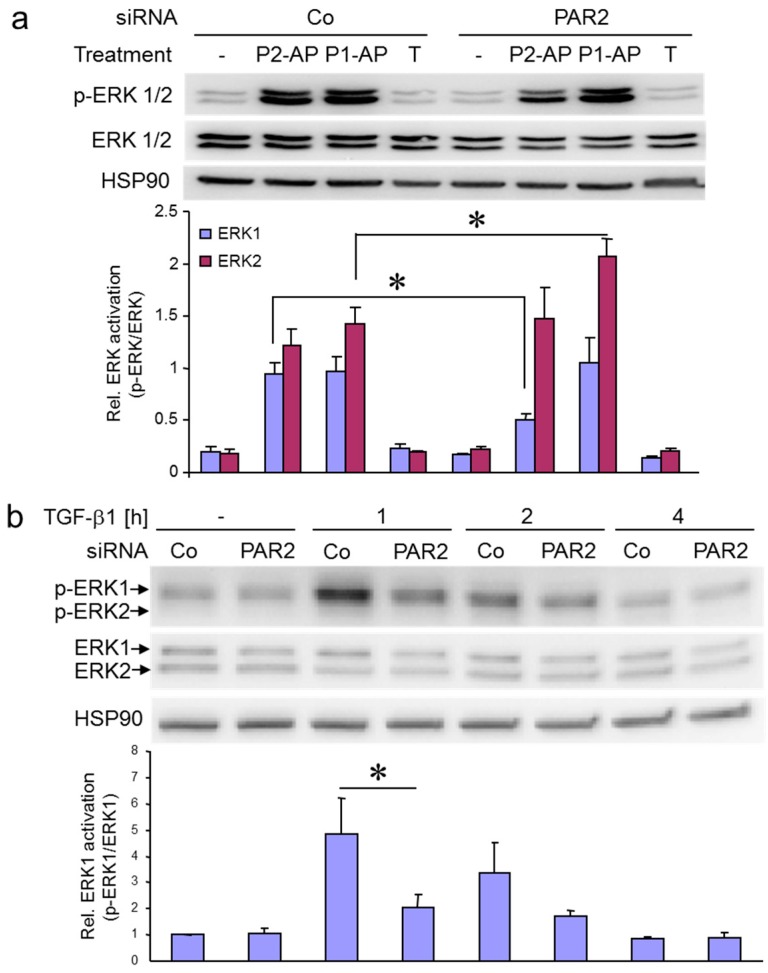
Both PAR2–AP- and TGF-β1-induced ERK activation are dependent on PAR2 protein expression. (**a**) Panc1 cells were transiently transfected with 50 nM of either control (Co) siRNA or siRNA specific to PAR2 (PAR2). Following stimulation with PAR2–AP (P2-AP), PAR1–AP (P1-AP) for 5 min or TGF-β (T) for 1 h, cells were subjected to immunoblotting for p-ERK1/2 and ERK1/2, and for HSP90 as a loading control. The graph below the blot shows densitometric data (mean ± SD) of underexposed bands derived from three parallel wells. One representative experiment is shown out of three performed in total. Asterisks indicate significance *p* < 0.05; (**b**) HaCaT cells were transfected with 50 nM of either Co siRNA or PAR2 siRNA, stimulated for the indicated times with TGF-β1, and processed for immunoblotting of p-ERK1/2 and ERK1/2. The graphs below the blots show results from densitometry-based quantification of three experiments, mean ± SD. The asterisk indicates significance *p* < 0.05.

To test whether the rapid activation of endogenous ERK1/2 in response to PAR2–AP in Panc1 cells was indeed PAR2 mediated, we knocked down the endogenous PAR2 protein by siRNA. To this end, depletion of PAR2 by siRNA in Panc1 cells attenuated PAR2–AP (2-fLI)-induced activation of ERK1 but not ERK2 (Figure 4a, lanes 2 vs. 6). However, the same cells were more responsive relative to control cells to stimulation with PAR1–AP (TFLLRN-NH_2_) (Figure 4a, lanes 3 vs. 7), while stimulation with TGF-β1 failed to increase p-ERK1 or pERK2 levels after 1 h of stimulation (Figure 4a, lanes 4 vs. 8). The only partial inhibitory effect of PAR2 siRNA on PAR2–AP-mediated ERK activation may be explained by heterodimer formation between PAR2 and PAR1 or by non-specific activation of PAR1 by PAR2–AP.

Given on the one hand the earlier demonstration of a requirement of PAR2 protein expression for the TGF-β1 pro-migratory and pro-invasive response in vitro [24], and on the other hand the crucial role of ERK activation in TGF-β1-induced cell migration (Ref. [11] and this study, Figure 1), the question arises as to whether ERK activation by TGF-β1 is PAR2 dependent. Available data indicate that PAR2 promotes TGF-β1-induced activation of SMAD2/3C and p38 MAPK [24]. However, its effect on other non-SMAD signaling pathways (e.g., MEK–ERK) has not yet been analyzed. Intriguingly, PAR2 depletion by siRNA strongly interfered with upregulation of p-ERK1 levels by TGF-β1 at 1 h of treatment (Figure 4b). A similar effect was observed in the PDAC cell line IMIM-PC1. The observation that TGF-β1-induced ERK1 activation is PAR2 dependent is a novel and unexpected finding.

### 2.5. Treatment with High Concentrations of GB88 Increases Basal and TGF-β1-Induced ERK Activation and Cell Migration

**Figure 5:**
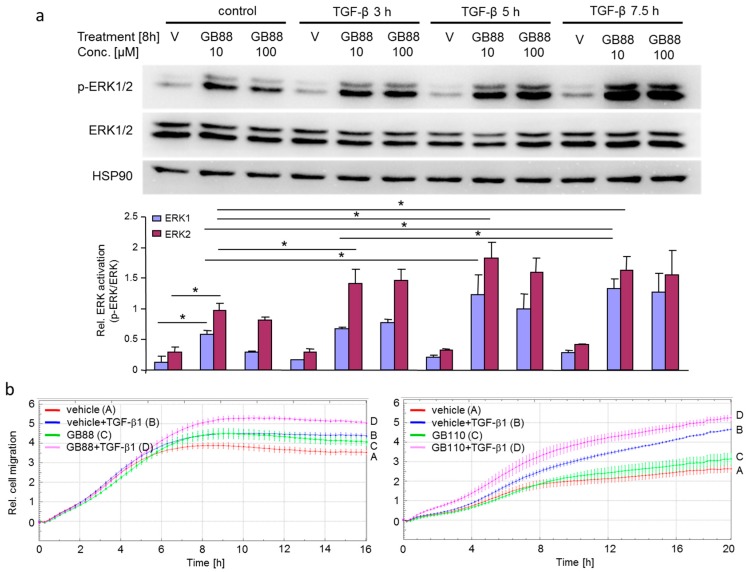
GB88 increases basal and TGF-β1-induced ERK activation and cell migration. (**a**) Panc1 cells were serum starved (1% fetal bovine serum, FBS) for 20 h prior to treatment for the indicated times with vehicle (V) or GB88 at concentrations (conc.) of either 10 µM or 100 µM. Crude cellular lysates were immunoblotted for p-ERK1/2, ERK1/2, and HSP90, and underexposed replicas subjected to densitometric analysis. Data in the graph represent the mean ± SD of three bands derived from cells from three parallel wells. One representative experiment is shown out of three performed in total. Asterisks indicate significance *p* < 0.05; (**b**) Panc1 cells were subjected to real-time cell migration assays in the presence of vehicle (0.1% DMSO) and either 10 µM of GB88 (left-hand graph) or GB110 (right-hand graph). In the left-hand graph, differences are significant (*p* < 0.05, unpaired Student’s *t*-test) between vehicle + TGF-β1 treated cells (blue curve, tracing B) and GB88 + TGF-β1 treated cells (magenta curve, tracing D) at 8:00 and all later time points. In the right-hand graph, differences are significant between vehicle + TGF-β1 treated cells (blue curve, tracing B) and GB110 + TGF-β1 treated cells (magenta curve, tracing D) at 4:00 and all later time points. In each graph, data are shown from one representative experiment out of three experiments performed in total.

In a recent study, we observed that treatment of Colo357 cells with 10 µM of the non-peptidic PAR2 biased agonist GB88 [33,34] stimulated rather than inhibited TGF-β1-dependent cell migration (Ref. [26], Figure 3b), although earlier work has shown that TGF-β1-induced cell migration requires PAR2 protein expression [24]. Interestingly, while GB88 inhibits G_q_-calcium signaling by PAR2, it has been shown to be an agonist in activating three other PAR2-derived pathways (cAMP, ERK, Rho) in human cells [33,34]. Given the crucial role of ERK in TGF-β1-mediated cell migration (see Figure 1), the synergistic increase in TGF-β1-dependent chemokinetic activity in the presence of 10 µM GB88 may have resulted from enhanced ERK activation. To this end, treatment of cells with GB88 alone (without TGF-β1) for 7.5 h at 10 or 100 µM GB88 increased p-ERK1/2 levels in Panc1 cells relative to vehicle-treated controls (Figure 5, lanes 1–3). Remarkably, combined treatment of Panc1 cells with 10 µM GB88 plus TGF-β1 (lanes 5, 8, and 11) synergistically and dramatically enhanced p-ERK1 levels in a time-dependent manner after 5 and 7. 5 h and p-ERK2 levels after 3, 5, and 7.5 h relative to 10 µM GB88/non-TGF-β1-treated controls (Figure 5a, lane 2). Similar results were obtained when GB88 was replaced with the potent non-peptidic PAR2 agonist GB110 [33,34] as control (Supplementary Figure S1).

Next, we tested the effect of GB88 and GB110 on cell migration induced by TGF-β1. As shown in Figure 5b, both GB88 (left-hand graph) and GB110 (right-hand graph), when used in combination with TGF-β1, further enhanced the migratory activity afforded by TGF-β1 alone.

### 2.6. PAR2 and ALK5 Can Be Co-Immunoprecipitated

**Figure 6:**
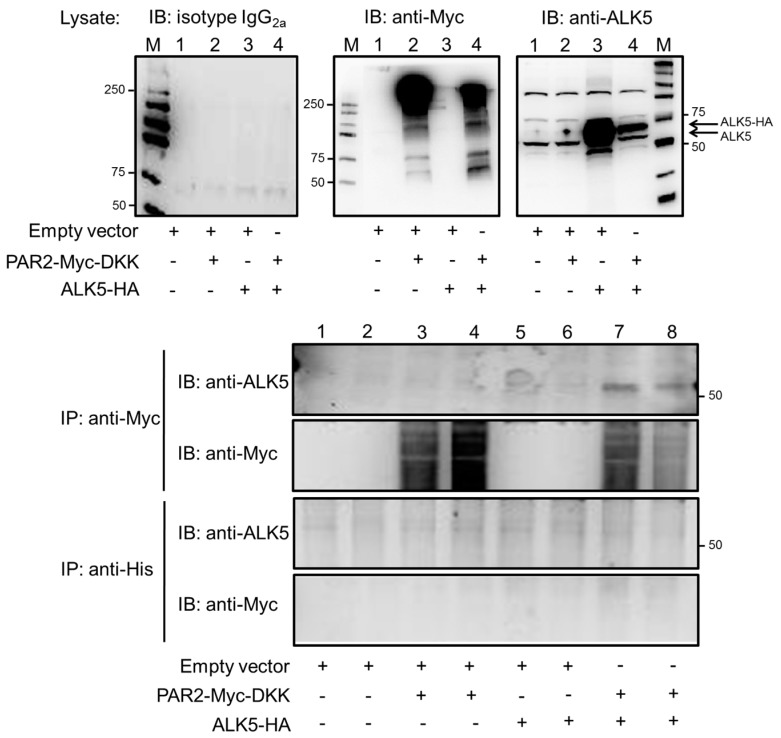
PAR2 and activin receptor-like kinase 5 (ALK5) can be co-immunoprecipitated. Panc1 cells were transfected with empty vector, PAR2-Myc-DKK, or ALK5-HA, alone or in combination, as indicated. Two days later, total cell lysates (20 µg each) were sequentially immunoblotted (IB) with IgG_2a_ as negative control (left blot), and with anti-Myc tag (middle blot) and anti-ALK5 antibodies (right blot) for detection of transfected PAR2 and transfected (and endogenous) ALK5 protein, respectively. The appearance of bands for endogenous ALK5 indicates equal protein loading. Subsequently, anti-Myc or anti-His microbead-based IP was used on ~1 mg of lysate (duplicate samples) to precipitate PAR2–Myc–DKK along with associated proteins followed by immunoblotting for ALK5 (upper panel) and anti-Myc tag (lower panel). Numbers next to the molecular weight marker (M) lanes indicate the molecular mass (in kDa). Note that PAR2–Myc–DKK migrates as a complex of diffuse bands between ~100 and >250 kDa because of heavy glycosylation [35,36]. Data shown are from a representative experiment out of five experiments performed in total.

Data from above indicate that PAR2 protein is partially required for TGF-β1-induced activation of ERK1/2 in HaCaT cells. PAR2 has been shown to couple to various other receptors of different classes [15] by receptor transactivation. Receptor transactivation may or may not involve physical interactions between both receptors [15]. Since both PAR2 and the TGF-β receptors reside at the cell surface from where they signal, we envisioned that PAR2 can physically interact with the TGF-β receptors to augment their activity. To test this possibility, we performed co-immunoprecipitation (co-IP) experiments following cotransfection of expression vectors encoding tagged versions of either PAR2 (PAR2–Myc–DKK) or ALK5 (ALK5–hemagglutinin (HA) in pcDNA3) into Panc1 cells. Panc1 cells were chosen because upon PAR2 depletion these cells exhibit a clear phenotype for all of the TGF-β responses we have analyzed [24,26]. Forty-eight hours after transfection, cells were lysed and tested by immunoblotting for successful transfection of PAR2–Myc–DKK and ALK5–HA. Only treatment with anti-Myc and anti-ALK5 antibodies, but not isotype IgG used as a control, yielded bands of the predicted size (Figure 6, upper three blots). Subsequently, lysates were subjected to anti-Myc or anti-His microbead-based co-IP followed by immunoblot analysis for ALK5. The resulting immunoblots showed a clear signal with the anti-ALK5 antibody only in anti-Myc immunoprecipitates but not in anti-His immunoprecipitates. Moreover, within the anti-Myc precipitated samples, bands were present only in those from PAR2–Myc–DKK/ALK5–HA double-transfectants but not from single transfectants or mock-transfected controls (Figure 6, lower two blots).

Taken together, our data indicate that PAR2 cooperates with TGF-β/ALK5 for cell migration and ERK activation via a mechanism that requires the expression of PAR2 [24] and is independent of PAR2’s ability to trigger calcium signaling [26]. In addition, our results argue in favor of a constitutive physical interaction between PAR2 and ALK5 in this cooperative transactivation mechanism.

## 3. Discussion

The effects of ERK on TGF-β signaling in pancreatic epithelial cells have been shown to be biphasic. P-ERK initially facilitates both TGF-β-induced cell cycle arrest and EMT but as cells begin to undergo transformation, TGF-β and ERK diverge with respect to the cell cycle. In contrast, ERK still drives TGF-β-induced EMT, converting TGF-β-induced activation of ERK as a mediator of tumor-suppressive TGF-β signals in normal pancreas epithelial cells towards a tumor promoter in the disease state [9]. ERK activation is also stimulated by PAR2 in response to activation by serine proteinases, activating peptides [13,14,15], and certain biased agonists (GB88, AY254) [16,17,33,34]. Tissue factor (TF)/Factor VIIa (FVIIa) has been shown to promote cell migration via PKCα and ERK-dependent c-Jun/AP-1 pathway in the colon cancer cell line SW620 [23] and in hepatocellular carcinoma progression [30]. Our own studies have shown that tumor cell-derived extracellular vesicles from malignant effusions, which present TF on their surface, can induce migration of Colo357 cells, which was sensitive to PAR2 and ERK inhibition [31]. Ras–MEK–ERK signaling is crucial for TGF-β-mediated cell migration [7,11,12]. Activation of ERK1/2 by TGF-β is more heterogeneous with respect to cell-type specificity and effective dose and more complex with respect to the molecular mechanisms involved. ERK can be triggered similar to tyrosine kinase receptors through the tyrosine kinase domain of ALK5, ShcA, Sos2, and Ras since ALK5 is a dual-specific kinase [8]. Alternatively, ERK activation involves transactivation of tyrosine kinase receptors. For instance, TGF-β1 induces EMT by the transactivation of EGFR/EGF signaling through HA/CD44 in lung and breast cancer cells [27].

In this study, we employed mainly PDAC-derived cells. Some experiments were complemented with results from HaCaT cells as these cells are highly responsive to TGF-β and have already been utilized for analysis of PAR2-induced ERK activation [28]. Since the effect of U0126 on PAR2–AP-induced cell migration in Colo357 cells and on TGF-β1-induced cell migration in Panc1 cells has been described by us [31] and others [11], respectively, we employed here the reverse combination, Panc1 cells for the PAR2–AP and Colo357 cells for TGF-β1 stimulation experiments. Results in Figure 1 show that ERK activation is crucial for the motility response to both PAR2–AP and TGF-β1 since migration was blocked by the MEK inhibitor U0126. Moreover, we found that both PAR2–AP and TGF-β1 can induce ERK activation in PDAC-derived cells and HaCaT keratinocytes. While stimulation of Panc1 and HaCaT cells with PAR2–AP generated a rapid increase in p-ERK levels (peak levels at 2–5 min), ERK activation in Panc1 and Colo357 cells challenged with TGF-β1 was delayed (peak levels between 1 and 12 h, depending on the cell type, see Figure 2). Despite the different kinetics, ERK activation in all three cell lines was independent of SMAD activation (see Figure 3). The most intriguing finding of this study, however, is the observation that the TGF-β1-induced ERK activation in HaCaT cells was dependent on the cellular expression of PAR2 protein (see Figure 4b), as was TGF-β1-induced cell migration and invasion in PDAC-derived cells [24,25]. Given the crucial role of MEK–ERK in TGF-β1-induced cell migration (see Figure 1), these findings also provide a likely explanation for the amplifying effect on this process seen upon treatment of the cells with 10 or 100 µM GB88. On its own, GB88 can increase the levels of p-ERK1/2 and in combination with TGF-β1 can synergistically enhance ERK1 and ERK2 phosphorylation, as well as cell migration (Figure 5). The stimulatory effects of GB88 on ERK activation and cell migration are likely independent of SMAD signaling since neither inhibition of ERK activation by U0126 (see Figure 3) nor treatment with 10 or 100 µM GB88 [26] was able to alter basal and TGF-β1-dependent SMAD3C phosphorylation. These results suggest that biased signaling by PAR2 enhances migration triggered by TGF-β1 [26].

TGF-β/ALK5 transactivation of PAR2 with subsequent ERK activation is a unique observation. As demonstrated by co-immunoprecipitation experiments with tagged versions of ALK5 and PAR2, this interaction may involve a direct or indirect physical interaction between the two receptors. Previously, only the “reverse” relationship—PAR2–AP-mediated transactivation of ALK5 with subsequent activation of SMAD2—has been reported [29]. In order to promote TGF-β1/ALK5-induced migration, ERK may be activated directly by PAR2 given the physical interaction between ALK5 and PAR2. However, it is also possible that ERK activation is initiated by the tyrosine kinase domain of ALK5 and that PAR2 promotes ERK activation in an indirect fashion by sustaining the expression of ALK5 [24]. The TGF-β–PAR2-dependent ERK signaling, however, is unlikely to promote cell migration via an enhancement of SMAD activation, since C-terminal SMAD2/3 phosphorylation is affected neither by ERK inhibition via U0126 (see Figure 3) nor by treatment with GB88 [26].

Based on the requirement of PAR2 for TGF-β1-dependent migration and invasion, we have postulated earlier that combined treatment for cancer patients with inhibitors of TGF-β/ALK5 and PAR2 signaling may be more effective than a single treatment regimen. However, the currently available PAR2 inhibitors (such as GB88) may not be suitable for this purpose because of their biased agonism. In particular, their ability to stimulate activation of ERK may disqualify them as anti-invasive or anti-metastatic agents for PDAC patients.

## 4. Materials and Methods

### 4.1. Reagents and Antibodies

The MEK inhibitor U0126 and the Rac1 inhibitor NSC23766 were purchased from Calbiochem/Merck. GB88 and GB110 were a kind gift of Dr. David B. Fairlie (The University of Queensland, Brisbane, Australia) [33,34]. The PAR2-selective peptide agonist SLIGKV-NH_2_ and the PAR1-selective agonist peptide TFLLRN-NH_2_ (STRAP-1) were obtained from Bachem (Bubendorf, Switzerland). Synthesis, coupling, cleavage from the resin and characterization of the PAR2-selective peptide 2-furoyl-LIGRLO-NH_2_ (2f-LI, EC_50_ = 2.5 μM) were done as described in detail before [26]. The following primary antibodies were used: anti-ALK5 antibody (TGFβ RI (V22), Santa Cruz Biotechnology, Santa Cruz, CA), anti-phospho-ERK1/2 (#4370, Cell Signalling Technology, Frankfurt/Main, Germany), anti-HSP90 (both #sc-7947 and #sc-13119), anti-ERK1/2 (#AF1576, R&D Systems, Wiesbaden, Germany), and anti-HA (clone 12CA5, Roche, Mannheim, Germany). HRP-linked anti-rabbit (#7074), anti-mouse (#7076), and anti-rat (#7077) secondary antibodies were from Cell Signaling Technology. The rhTGF-β1 (#300-023) was purchased from ReliaTech (Wolfenbüttel, Germany) and used at a concentration of 5 ng/mL.

### 4.2. Cell Culture

The established human pancreatic carcinoma cell lines Panc1 and Colo357 were originally purchased from the ATCC and routinely maintained in RPMI 1640 (Lonza, Basel, Switzerland) supplemented with 10% FBS, 2 mM l-glutamine, 2 mM sodium pyruvate, and penicillin/streptomycin. Another TGF-β-responsive PDAC line, IMIM-PC1, a kind gift from Dr. A. Menke (University of Giessen, Giessen, Germany), was cultured as Panc1 cells. The immortalized human keratinocyte cell line HaCaT was cultured in DMEM and the same supplements. All cells were routinely tested for the absence of mycoplasma contamination.

### 4.3. Transient Transfection of siRNAs

The siRNAs to PAR2 or ALK5 (in each case a pool of three validated Stealth siRNAs), a Stealth siRNA Negative control (all purchased from Life Technologies, Carlsbad, CA) were introduced by serum-free transfection using Lipofectamine RNAiMAX (Life Technologies) according to the manufacturer’s instructions. Twenty-four hours later cells underwent another round of transfection and after another 48 h were processed for Western blot analysis of p-ERK1/2 and ERK1/2.

### 4.4. Immunoblot Analysis and Co-Immunoprecipitation

For immunoblot analysis, cells were lysed in RIPA buffer and the protein concentrations determined by Bradford assay (Bio-Rad, Hercules, CA, USA). Equal amounts of protein were fractionated by SDS-PAGE and transferred to PVDF membrane. Following cotransfection of Panc1 cells with expression vectors for Myc–DKK-tagged PAR2 (Origene, Rockville, MD, USA) and HA-tagged ALK5, PAR2 protein was co-immunoprecipitated with anti-Myc microbeads (µMACS c-Myc Isolation Kit, #130-091-123) or anti-His microbeads (#130-091-124) as control from cellular lysates of Panc1 cells according to the protocol provided by the supplier (Miltenyi Biotec, Bergisch-Gladbach, Germany) and subsequently analyzed by immunoblotting with anti-ALK5 antibody.

### 4.5. Migration Assays

Cell migration (chemokinesis) of Panc1 and Colo357 cells was measured with the xCELLigence^®^ DP system (ACEA Biosciences, San Diego, CA, USA). Cells were serum starved (RPMI + 0.5% FBS) for 24 h prior to the assay, while the assays were run in the presence of RPMI with 1% FBS. Inducers of migration, TGF-β1-induced, were added together with the cells (40,000 cells/well) to the wells of the upper chamber and without cells to the lower chamber of the CIM plates—16 according to previous descriptions [24,26] and the instruction manual (chemokinesis setup). In addition, we performed assays in which TGF-β1 or PAR2–AP was added only to the lower chamber (chemotaxis setup). Prior to plate assembly, the bottom side of the upper chamber was coated with collagen I (30 μL of a 400 μg/mL solution). Migratory activity reflected by the dimensionless cell index was recorded every 15 min for up to 30 h using the RTCA software (version 1.2, ACEA Biosciences).

### 4.6. Statistical Analysis

Statistical significance was calculated using the unpaired Student’s *t*-test. Results were considered significant at *p* < 0.05 (*).

## Supplementary Materials

Supplementary materials can be found at www.mdpi.com/1422-0067/18/12/2776/s1.

## Abbreviations

ALK5	Activin receptor-like kinase 5
AP	Agonistic peptide
ERK	Extracellular signal-regulated kinase
PAR2	Proteinase-activated receptor 2
TGF-β	Transforming growth factor-β
