# Supplementary material for: The Role of PAR2 in TGF-β1-Induced ERK Activation and Cell Motility
Source: Int J Mol Sci. 2017 Dec 20;18(12):2776. doi: 10.3390/ijms18122776 (PMC5751374; doi:10.3390/ijms18122776)
Supplement: Supplementary file 1 [file ijms-18-02776-s001.pdf]

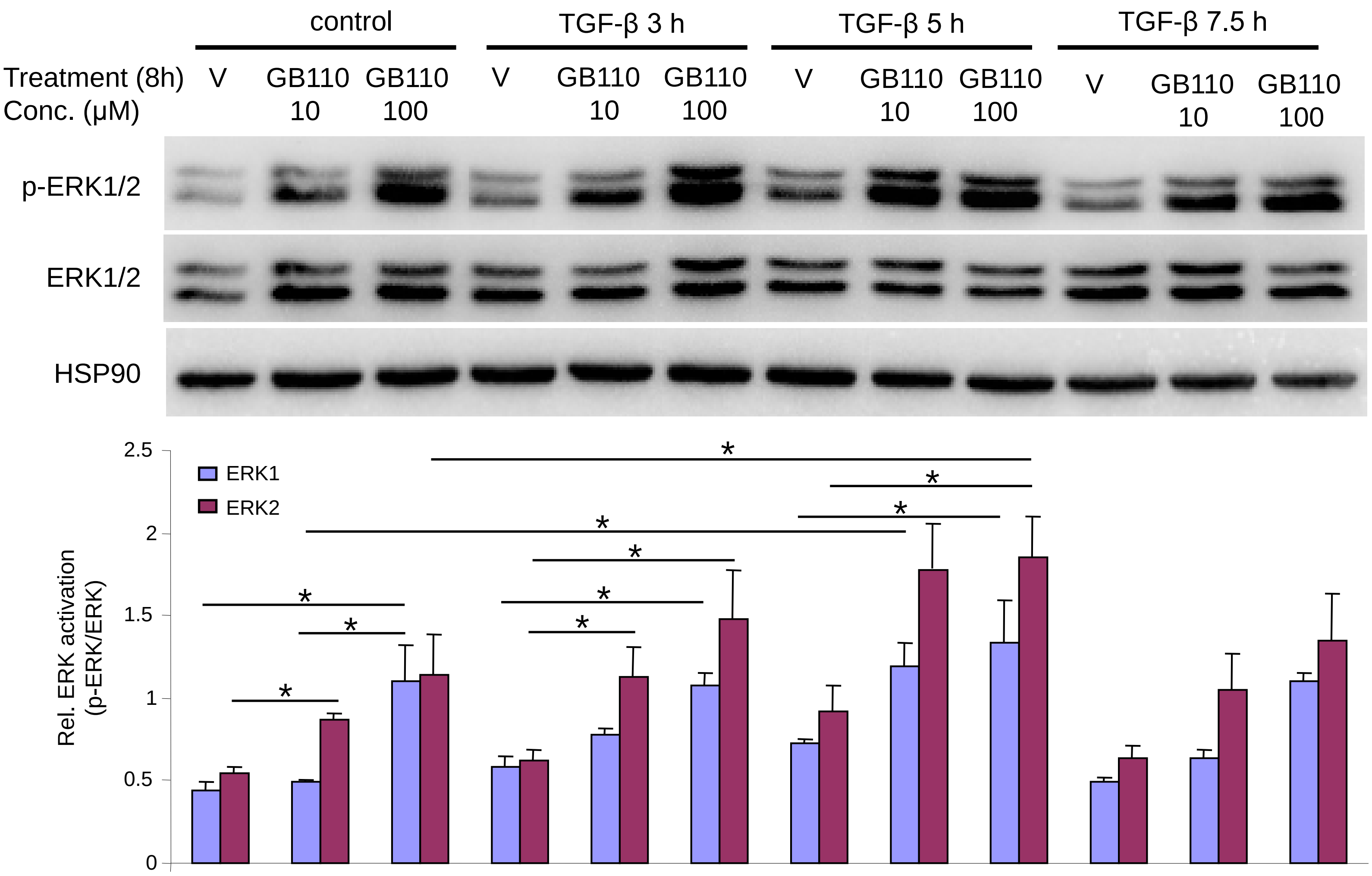

Supplementary Figure S1. GB110 increases basal and TGF-β1-induced ERK activation. Panc1 cells were serum-starved (1% FBS) for 20 h prior to treatment for the indicated times with vehicle (V) or GB110 at concentrations (conc.) of either 10 µM or 100 µM. Crude cellular lysates were immunoblotted for p-ERK1/2, ERK1/2, and HSP90, and underexposed replicas subjected to densitometric analysis. Data in the graph represent the mean ± SD of three bands derived from cells from three parallel wells. One representative experiment is shown out of three performed in total. Asterisks indicate significance (p<0.05).
